# LOC401317, a p53-Regulated Long Non-Coding RNA, Inhibits Cell Proliferation and Induces Apoptosis in the Nasopharyngeal Carcinoma Cell Line HNE2

**DOI:** 10.1371/journal.pone.0110674

**Published:** 2014-11-25

**Authors:** Zhaojian Gong, Shanshan Zhang, Zhaoyang Zeng, Hanjiang Wu, Qian Yang, Fang Xiong, Lei Shi, Jianbo Yang, Wenling Zhang, Yanhong Zhou, Yong Zeng, Xiayu Li, Bo Xiang, Shuping Peng, Ming Zhou, Xiaoling Li, Ming Tan, Yong Li, Wei Xiong, Guiyuan Li

**Affiliations:** 1 Hunan Cancer Hospital and the Affiliated Cancer Hospital of Xiangya School of Medicine, Central South University, Changsha, Hunan, China; 2 Key Laboratory of Carcinogenesis of Ministry of Health and Key Laboratory of Carcinogenesis and Cancer Invasion of Ministry of Education, Cancer Research Institute, Central South University, Changsha, Hunan, China; 3 Department of Oral and Maxillofacial Surgery, the Second Xiangya Hospital, Central South University, Changsha, Hunan, China; 4 Department of Stomatology, Xiangya Hospital, Central South University, Changsha, Hunan, China; 5 Hunan Key Laboratory of Nonresolving Inflammation and Cancer, Disease Genome Research Center, The Third Xiangya Hospital, Central South University, Changsha, Hunan, China; 6 Department of Laboratory Medicine and Pathology and Masonic Cancer Center, University of Minnesota, Minneapolis, Minnesota, United States of America; 7 Mitchell Cancer Institute, University of South Alabama, Mobile, Alabama, United States of America; 8 Department of Biochemistry and Molecular Biology, School of Medicine, University of Louisville, Louisville, Kentucky, United States of America; CSIR Institute of Genomics and Integrative Biology, India

## Abstract

Recent studies have revealed that long non-coding RNAs participate in all steps of cancer initiation and progression by regulating protein-coding genes at the epigenetic, transcriptional, and post-transcriptional levels. Long non-coding RNAs are in turn regulated by other genes, forming a complex regulatory network. The regulation networks between the p53 tumor suppressor and these RNAs in nasopharyngeal carcinoma remains unclear. The aims of this study were to investigate the regulatory roles of the *TP53* gene in regulating long non-coding RNA expression profiles and to study the function of a *TP53*-regulated long non-coding RNA (LOC401317) in the nasopharyngeal carcinoma cell line HNE2. Long non-coding RNA expression profiling indicated that 133 long non-coding RNAs were upregulated in the human NPC cell line HNE2 cells following *TP53* overexpression, while 1057 were downregulated. Among these aberrantly expressed long non-coding RNAs, LOC401317 was the most significantly upregulated one. Further studies indicated that LOC401317 is directly regulated by p53 and that ectopic expression of LOC401317 inhibits HNE2 cell proliferation *in vitro* and *in vivo* by inducing cell cycle arrest and apoptosis. LOC401317 inhibited cell cycle progression by increasing p21 expression and decreasing cyclin D1 and cyclin E1 expression and promoted apoptosis through the induction of poly(ADP-ribose) polymerase and caspase-3 cleavage. Collectively, these results suggest that LOC401317 is directly regulated by p53 and exerts antitumor effects in HNE2 nasopharyngeal carcinoma cells.

## Introduction

Nasopharyngeal carcinoma (NPC) is one of the most common malignant head and neck tumors and initiates in the nasopharyngeal epithelium [1]–[5]. High incidences of NPC are observed in Southeast Asia and southern China, resulting in serious healthcare problems in these regions [6]–[8]. Radiotherapy has been used as the primary clinical treatment for all stages of NPC over the last several decades in China [9]–[11].

The *TP53* gene encodes the p53 protein and is an important tumor suppressor [12], [13]. It is estimated that over 50% of human tumors harbor mutations in the *TP53* gene and the majority of tumors have dysfunctional p53 signaling [14]–[16]. p53 participates in all steps of tumor initiation and development by regulating the expression of many downstream genes; thus, p53 is also an important candidate target for cancer gene therapy [16], [17].

The dysfunction of *TP53* was also demonstrated to closely correlate with NPC initiation and development. Pan *et al*. reported that the injection of the exogenous *TP53* gene into NPC patients significantly improved radiosensitivity [18]. Therefore, the restoration of p53 function or its downstream signaling pathways is potentially of great value in treating NPC patients.

Long non-coding RNAs (lncRNAs) comprise a large set of RNA molecules that exceed 200 nt in length, completely lack or possess limited protein-coding capacity, and represent a substantial portion of the transcriptome [19], [20]. lncRNAs widely regulate gene expression at the epigenetic, transcriptional, and post-transcriptional levels [21]–[24]. Substantial evidence indicates that the aberrant expression or dysfunctional activities of lncRNAs are correlated with tumor initiation and progression [25]–[28]. Recent studies have revealed that lncRNAs interact with the p53 pathway and form a complex regulatory network [29]–[33]. For example, using mouse embryo fibroblast (MEF) cells from p53 knockout mice, Huarte *et al*. found that lincRNA-p21 is regulated by p53 and governs the expression of hundreds of genes normally repressed by p53 [32]. Liu *et al*. showed that LOC285194 is a p53 transcription target, and that ectopic LOC285194 expression inhibits colorectal carcinoma cell (HCT116) growth, both *in vitro* and *in vivo* [33].

To investigate the regulatory roles of the *TP53* gene on the expression and functions of p53-regulated lncRNAs in NPC, we overexpressed the *TP53* gene in the NPC cell line HNE2 and monitored the resultant lncRNA expression profiles using an lncRNA microarray. The results indicated that a set of lncRNAs showed significantly aberrant expression in HNE2 cells following *TP53* overexpression. lncRNA LOC401317 was the most significantly upregulated lncRNA. Further studies indicated that LOC401317 is directly transcribed by p53 and that LOC401317 inhibits HNE2 cell growth by arresting cell cycling and inducing apoptosis, both *in vitro* and *in vivo*.

## Materials and Methods

### Ethics Statement

All animal procedures were carried out in strict accordance with the recommendations in the Guide for the Care and Use of Laboratory Animals of the Central South University. The Institutional Animal Care and Use Committee (IACUC) of Central South University (Changsha, Hunan, China) specifically approved this study, under Permit Number: 2012-28. All surgery was performed under sodium pentobarbital anesthesia, and all efforts were made to minimize suffering. Animals were allowed access to standard chow diet and water *ad libitum* and were housed in a pathogen-free barrier facility with a 12L: 12D cycle. Mice were sacrificed by CO_2_ asphyxiation.

### Plasmids

The p53 expression vector, pCMV-p53, was purchased from Clontech Laboratories Inc. (Mountain view, CA, USA). The pGL3-Enhancer luciferase reporter plasmid was purchased from Promega Corporation (Madison, WI, USA). The pp53-TA-luc plasmid, a luciferase reporter plasmid used for assaying p53 transcriptional activity, was purchased from the Beyotime Institute of Biotechnology (Shanghai, China). This plasmid contains multiple conserved p53-binding sites and was used for sensitive detection of p53 transcriptional activity. The pcDNA3.1 vector was purchased from Invitrogen (Carlsbad, CA, USA).

To construct the LOC401317 expression vector, the entire LOC401317 sequence [GenBank: XR_242178.1] was amplified by reverse transcriptase PCR (RT-PCR) using the forward primer 5′-ATGTATTGTAATCTTTGCTCAGTCC-3′ and the reverse primer 5′-TGACGTAATGGACCTGTATCC-3′ and cloned into pcDNA3.1. The putative LOC401317 promoter was PCR-amplified from human genomic DNA using the forward primer 5-′ACGCGTTGAAGAAGGGGTGACAAG-3′ and the reverse primer 5′-AAGCTTCCCTACTCCTTGCCCTGT-3′. The resulting 820-bp amplicon was then inserted 5′ of the luciferase gene in the PGL3-enhancer vector. In addition, three tandem wild-type or mutant p53-binding sites were inserted into the TA-luc vector, using synthetic oligonucleotides encoding a wild-type p53-binding site (5′-GGGACCGGGGCGGGGCCTGCCCCCT-3′) or a mutant p53-binding site (5′-AGGACCGGGGCGGGGTCTACCCCCT-3′).

### Cell culture and transfection

Low-passage human NPC HNE2, HNE1, and CNE2 [4] cells were maintained in RPMI-1640 media (Invitrogen, Carlsbad, CA, USA) supplemented with 10% fetal bovine serum (FBS; Invitrogen). Cells were grown at 37°C in a humidified 5% CO_2_ incubator. HNE2 were transfected with various plasmids using Lipofectamine 2000 (Invitrogen), according to the manufacturer's suggested protocol. To generate stable transfectants, transfected HNE2 cells were cultured in complete medium for 48 h and then selected for 3 weeks in medium containing 400 µg/ml G418 (Invitrogen). Then LOC401317 overexpression in stable transfectants was confirmed by quantitative real-time polymerase chain reactions qRT-PCR.

### RNA isolation and qRT-PCR

Total RNA was extracted from HNE2 cells using the TRIzol Extraction Kit (Invitrogen) and reverse transcribed using AMV reverse transcriptase (Promega). The mRNA expression levels of *TP53*, *MDM2*, *LOC401317* and 4 other lncRNAs were determined by qRT-PCR using the SYBR Green (Invitrogen) method with primers specific for each target mRNA: *TP53*, 5′-CCCTTCCCAGAAAACCTACC-3′ and 5′-CTCCGTCATGTGCTGTGACT-3′; *MDM2*, 5′-GTATCAGGCAGGGGAGAGTG-3′ and 5′-GAAGCCAATTCTCACGAAGG-3′; *LOC401317*, 5′-AGTCCAACGTGGTCCCTTC-3′ and 5′-AACTTTCTCCGGGGGTTC-3′; *SNHG12*, 5′-GACTTCCGGGGTAATGACAG-3′ and 5′-GCCTTCTGCTTCCCATAGAG-′3; *LINC00472*, 5′-CACTGGGCATTTTCTCTTCA-3′ and 5′-CCTATCCCTTTCCCTCTGCT-3′; *LOC100129931*, 5′-TACGCACAGATATGCCACCA-3′ and 5′-CGTTGTTCCTCCAGCTTCTT-3′; *LOC442028*, 5′-GCAACCACAAGGAGTTGAATG-3′ and 5′-CAGGCTTCTCAGTGCCAGAC-3′. Primers targeting *β-actin* mRNA (5′-GCATCCCCCAAAGTTCACAA-3′ and 5′-AGGACTGGGCCATTCTCCTT-3′) were used as internal controls.

### lncRNA expression profiling

Microarray analysis was performed by a commercial company (Oebiotech, Shanghai, China), using the OE Human lncRNA Microarray V2.0 (Oebiotech) that contains approximately 46,500 lncRNAs. Briefly, total RNA samples isolated from HNE2 cells at 0, 12, 24, or 48 h post-transfection with pCMV-p53 were reverse transcribed using random hexamers and oligo-dT primers. cRNAs were synthesized with T7 RNA polymerase and labeled utilizing Cy3-CTP, after which they were hybridized to the microarray [34]. Following hybridization and wash steps, the processed slides were scanned with an Agilent Microarray Scanner (Agilent Technologies, Santa Clara, CA, USA), and the acquired array images were analyzed using Agilent Feature Extraction Software (Agilent Technologies), which performs background subtractions. Quantile normalization and subsequent data processing were performed using the GeneSpring GX v. 11.0 software package (Agilent Technologies). All microarray expression data were deposited in the Gene Expression Omnibus database under Accession No. GSE60379. Threshold fold changes of >2 were used to screen for upregulated or downregulated lncRNAs.

### Luciferase assay

Luciferase assays were performed using the Dual-Glo Luciferase Assay Kit (Promega, Madison, WI, USA), according to the manufacturer's protocol. Briefly, cells were first transfected with appropriate plasmids in 24-well plates. After 0, 12, 24, or 48 h, cells were harvested, lysed in buffer containing luciferase substrate, and luciferase activities were measured using a microplate luminometer (Beckman Coulter, Fullerton, CA, USA) [35]. *Renilla* luciferase was used for normalization purposes.

### Western blot analyses

Equal amounts of protein were resolved on 10% SDS-PAGE gels, blotted onto nitrocellulose membranes, and incubated overnight at 4°C with primary antibodies in PBS containing 0.05% (vol/vol) Tween 20 (T-PBS) and 1% (wt/vol) bovine serum albumin. Membranes were incubated overnight at 4°C with primary antibodies. The primary antibodies used included the following: rabbit monoclonal antibodies against p53 (Cell Signaling Technology, Danvers, MA, USA), p21 (Cell Signaling Technology), cyclin D1 (Abcam, Cambridge, Massachusetts, USA), and α-tubulin (Abcam); mouse monoclonal antibodies against cyclin E1 (Cell Signaling Technology) and GAPDH (Cell Signaling Technology); rabbit polyclonal antibodies against MDM2 (Abcam), poly(ADP-ribose) polymerase (PARP; Cell Signaling Technology), and cleaved caspase-3 (Cell Signaling Technology). The blots were then washed in T-PBS, incubated with an HRP-conjugated secondary antibody, washed in T-PBS, and visualized using the ECL Western Blotting Substrate (Pierce, Rockford, IL, USA).

### MTT assay

The effect of LOC401317 expression on HNE2 cell growth was measured in MTT assays. Briefly, HNE2 cells stably transfected with the pcDNA3.1-LOC401317 expression vector or the parental pcDNA3.1 vector were seeded into 96-well plates at 1×10^3^ cells/well, and cell growth was assessed every 24 h. Cells were exposed to 3-(4,5-dimethylthiazol-2-yl)-2,5-diphenyltetrazolium bromide (MTT; 25 µl/well, 5 mg/ml; Sigma Aldrich, St. Louis, MO, USA) for 4 h. The formazan generated in each well was dissolved in 150 µl of dimethyl sulfoxide, and optical densities were measured at 490 nm.

### Flow cytometry analysis of cell cycle distribution and cell apoptosis

To measure cell cycle distributions, cells were collected, washed with PBS, and fixed in 70% (v/v) ethanol overnight. Cells were digested with 100 mg/ml of RNase A in PBS and stained in the dark for 30 min with 40 mg/ml of propidium iodide (Sigma Aldrich), followed by flow cytometry analysis. To measure apoptosis, cells were collected, washed with PBS, and stained with fluorescein isothiocyanate-labeled annexin V (Invitrogen) and propidium iodide, followed by flow cytometry analysis.

### Xenograft model

Male BALB/c nude mice at 4–6 weeks of age were used and divided into three groups (10 mice/group). To assess the effect of LOC401317 expression on tumorigenicity *in vivo*, 5×10^6^ stable HNE2 cell transfectants (pcDNA3.1-LOC401317 or the parental pcDNA3.1 vector) or untreated HNE2 cells (mock) were subcutaneously injected into the flanks of BALB/c mice. The lengths, widths, and depths of tumors were measured every 5 days. Mice were sacrificed at 5 weeks post-injection by CO_2_ asphyxiation. Tumor volumes were estimated using the following equation: volume  =  length (mm) × width (mm) × depth (mm) ×0.5.

### *In situ* hybridization

*In situ* hybridization (ISH) was used to detect human LOC401317 in xenograft tumor tissues. Two antisense sequences of human LOC401317 used for probes were as follows: 5′-GTAATGGACCTGTATCCAGTCTTTATTCCTTGAC-3′ and 5′-CACGTTGGACTGAGCAAAGATTACAATACA-3′. The sense probe was used as a negative control, while an anti-GAPDH probe was used as a positive control. Probes were labeled with DIG-dUTP on their 3′ ends and synthesized by Sangon Biotech Inc. (Shanghai, China). *In situ* hybridization was performed as previously described [36].

### Immunohistochemistry

Immunohistochemistry (IHC) was performed using the peroxidase-antiperoxidase technique after a microwave antigen retrieval procedure. Sections were incubated with mouse anti-human p21 (Cell Signaling Technology, Danvers, MA), cleaved caspase-3 (Cell Signaling Technology), cyclinD1 (Abcam, Cambridge, Massachusetts, USA), cyclinE1 (Cell Signaling Technology), or PARP (Cell Signaling Technology) antibodies overnight at 4°C. A semiquantitative scoring criterion was used, in which both staining intensities and positive areas were recorded. [37]

### Statistical analysis

Significant differences in gene expression levels, cell survival rates, and proliferation rates between groups were analyzed by ANOVA analysis, and results are expressed as mean ± standard deviations (SD). *P* values of <0.05 were considered statistically significant, and all statistical tests were two-sided.

## Results

### *TP53* overexpression in the NPC cell line HNE2

**Figure 1:**
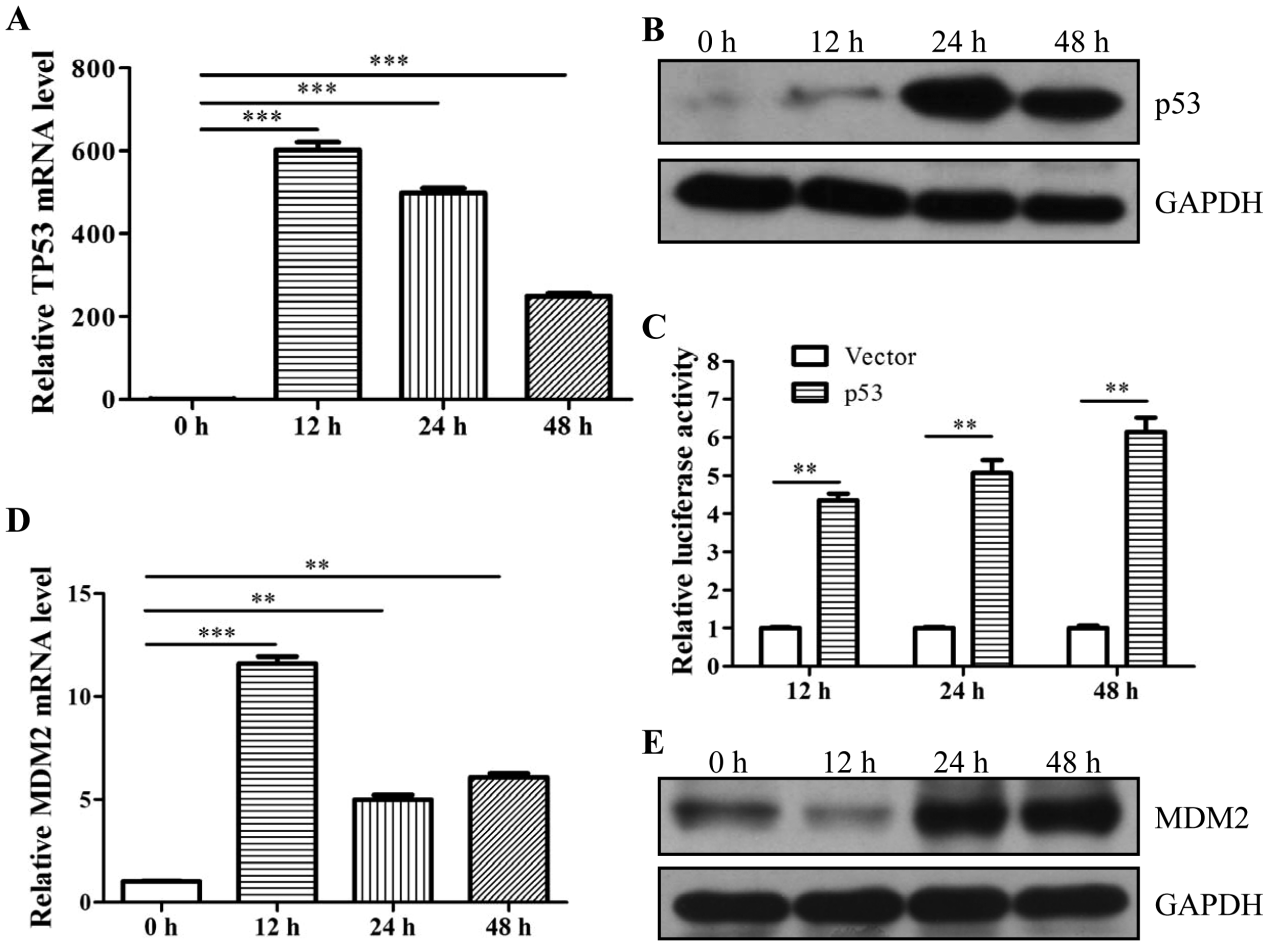
*TP53* overexpression in HNE2 cells. HNE2 cells were transfected with a *TP53* expression vector (the pCMV-p53 plasmid). Levels of (**A**) *TP53* mRNA transcripts and (**B**) p53 protein expression were determined at 0–48 h post-transfection by qRT-PCR and western blotting, respectively. (**C**) To measure p53 transcriptional activity, the pCMV-p53 and the pp53-TA-luc plasmids were cotransfected into HNE2 cells, and transcriptional activity of p53 from 0–48 h post-transfection was determined by luciferase assays. Similarly, induction of (**D**) MDM2 mRNA transcripts and (**E**) MDM2 protein expression in HNE2 were also determined in HNE2 pCMV-p53 transfectants by qRT-PCR and western blotting. GAPDH protein expression was detected as a loading control for p53 and MDM2 western blots. Data shown are representative of 3 independent experiments. Bar graphs show mean ± S.D. **P<0.01, ***P<0.001.

The *TP53* gene was overexpressed in a human NPC cell line by transfecting HNE2 cells with a pCMV-p53 plasmid. HNE2 cells were harvested at 0, 12, 24, and 48 h post-transfection, and the expression and transcriptional activity of p53 were determined by qRT-PCR (Figure 1A), western blotting (Figure 1B), and luciferase assays (Figure 1C). qRT-PCR results showed that *TP53* mRNA expression reached a maximum at 12 h post-transfection. Expression of the p53 protein was maximal at 24 h post-transfection and decreased subsequently, suggesting that p53 protein expression may be negatively regulated by endogenous feedback inhibition. The MDM2 protein is known to act as a critical negative regulator for p53; thus, we examined MDM2 expression levels 0, 12, 24, and 48 h post-transfection. Both MDM2 mRNA (Figure 1D) and protein (Figure 1E) expression levels were markedly increased at 12 h following *TP53* gene transfection. These data suggested that the p53 expression was limited due to feedback inhibition by MDM2.

### lncRNA expression profiles in HNE2 cell following *TP53* transgene expression

**Figure 2:**
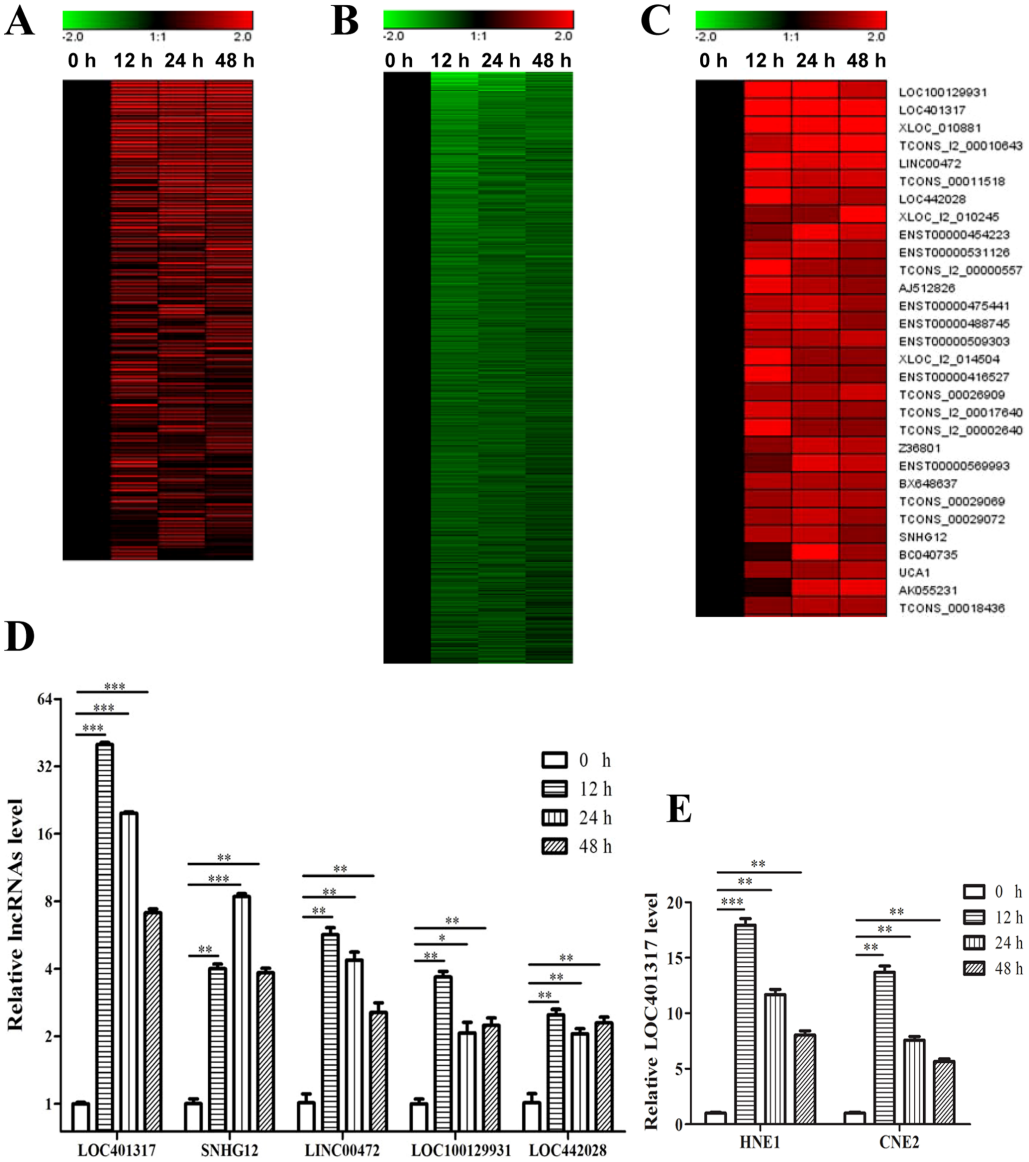
lncRNAs that are dysregulated by TP53 overexpression. (**A**) A total of 133 lncRNAs were upregulated in HNE2 cells by more than 2.0-fold in at least one time point (12, 24, or 48 h post-pCMV-p53 transfection), while (**B**) 1057 lncRNAs were downregulated by more than 2.0-fold in at least 1 time point in HNE2 cells. (**C**) Detailed expression profiles of the top 30 lncRNAs that were the most significantly upregulated by *TP53* transgene expression. (**D**) Validation of 5 of the top 30 most-upregulated lncRNAs in HNE2 by qRT-PCR. Data shown reflect the means of 3 independent experiments ± S.D. *P<0.05, **P<0.01, ***P<0.001. Expression data are exhibited as normalized log ratios to control time points (0 h) for each lncRNA. (**E**) Validation of LOC401317 expression in HNE1 and CNE2 cells following TP53 transfection by qRT-PCR. Data shown reflect the mean of 3 independent experiments ± S.D. **P<0.01, ***P<0.001. Expression data were normalized to control time points (0 h).

To identify *TP53*-regulated lncRNAs in NPC, we first harvested HNE2 cells at 0, 12, 24, and 48 h following transfection with the pCMV-p53 expression vector. Total RNA was reverse transcribed and the resulting cDNAs were hybridized to a human lncRNA microarray containing ∼46,500 lncRNAs. lncRNA expression profiling revealed 133 lncRNAs that were upregulated by ≧2.0-fold (Figure 2A) and another 1057 lncRNAs that were downregulated by ≧2.0-fold (Figure 2B) in pCMV-p53 transfectants in at least 1 time point. Detailed information on dysregulated lncRNAs and their expression data is shown in Table S1. To validate lncRNA microarray findings, we selected 5 lncRNAs from the top 30 lncRNAs that were the most significantly upregulated (Figure 2C) and confirmed that their expression had increased by qRT-PCR (Figure 2D). The results confirmed that LOC100129931, LOC401317, LINC00472, LOC442028, and SNHG12 were overexpressed in HNE2 cells following *TP53* overexpression (*P*<0.05 in each case), consistent with the microarray data. Among these 5 lncRNAs, LOC401317 was confirmed as the most significantly upregulated lncRNA by qRT-PCR. The upregulation of LOC401317 following *TP53* overexpression was confirmed in 2 additional NPC cell lines (HNE1 and CNE2) by qRT-PCR (Figure 2E). However, no studies in the literature have discussed the functional role(s) of lncRNA LOC401317 thus far, so its function(s) in NPC carcinogenesis is completely unknown. Thus, we chose LOC401317 for functional analysis to provide insights into its potential role(s) in NPC.

### Direct transcription of LOC401317 by p53

**Figure 3:**
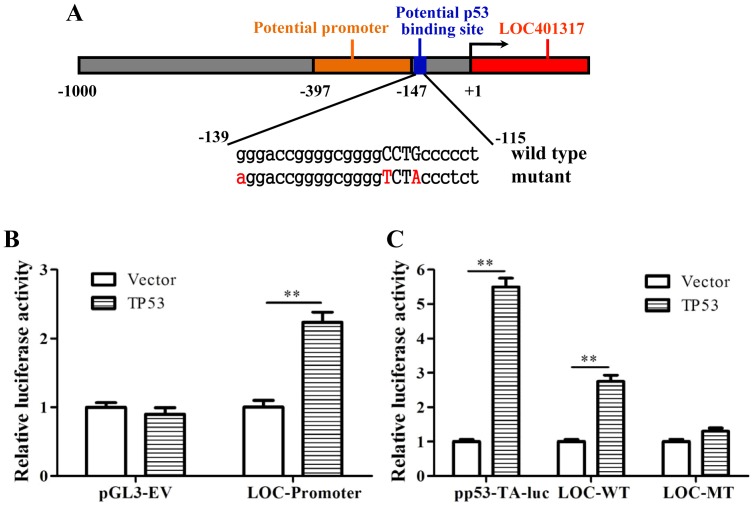
p53 transcriptionally upregulates LOC401317 through a p53-binding site upstream of the LOC401317 transcription start site. (**A**) Schematic representation of the potential promoter and p53-binding site upstream of LOC401317 transcription start site. Wild-type and mutated versions of the putative p53-binding site are indicated. (**B**) Transactivation of the putative LOC401317 promoter by p53. An 820-bp sequence, −570 bp to +249 bp to the transcript start site of LOC401317, containing the potential promoter and p53-binding site was inserted into the pGL3 luciferase reporter vector (LOC-promoter). The empty pGL3 vector served as a negative control (pGL3-EV). Luciferase vectors were cotransfected with pCMV-p53 or the parental vector (Vector) into HNE2 cells, as indicated. Cells were then harvested for luciferase assays 24 h after transfection. (**C**) Activation of the potential p53-binding site adjacent to the LOC401317 promoter by p53. Synthetic oligonucleotides containing 3 tandem wild-type p53-binding sites (LOC-WT) or mutant p53-binding sites (LOC-MT, negative control) were inserted into the luciferase reporter vector. The pp53-TA-luc vector, which contains conserved p53-binding sites, was used as a positive control. Luciferase vectors were cotransfected with pCMV-p53 or the parental vector into HNE2 cells, as shown. Cells were harvested for luciferase assays 24 h after transfection. The results showed that p53 significantly induces luciferase activity (>2.76-fold) via the wild-type p53-binding site. Data shown are the means of 3 independent experiments ± S.D. **P<0.01.

p53 is well known to exert its tumor suppressive function by serving as a transcription factor. To determine whether p53 transcriptionally upregulates LOC401317, the online Promoter Scan (www-bimas.cit.nih.gov/molbio/proscan) and Genomatix (www.genomatix.de/) programs were used to analyze the 1000-bp sequence upstream of the transcription start site of LOC401317. A potential promoter was predicted at −397 to −147 bp relative to the transcript start site of LOC401317. A putative p53-binding site spanned the −139 to −115 bp positions, adjacent to the potential promoter region (Figure 3A). Thus, we cloned an 820 bp (−570 bp to +249 bp) fragment containing the potential promoter and p53-binding site into the pGL3 luciferase reporter vector, which was then cotransfected with pCMV-p53 into HNE2 cells. Luciferase assays indicated that p53 induced luciferase activity by over 2.2-fold, while no effect was observed with an empty pGL3 vector control (Figure 3B). To confirm that p53 transcriptionally regulates LOC401317 through the predicted p53-binding site, synthetic oligonucleotides containing 3 tandem wild-type and mutant p53-binding sites (sequences shown in Figure 3A) were inserted into the luciferase reporter vector. The pp53-TA-luc vector containing conserved p53-binding sites was used as a positive control. We found that p53 overexpression significantly induced luciferase activity (>2.7-fold) in cells transfected with a reporter vector containing the wild-type putative promoter sequence of the predicted p53 binding in the LOC401317 gene (Figure 3C).

### LOC401317 inhibits HNE2 cell growth by inducing cell cycle arrest and apoptosis, both *in vitro* and *in vivo*

**Figure 4:**
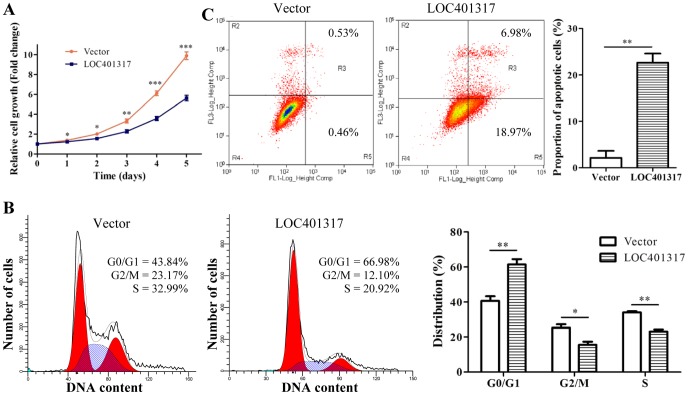
LOC401317 inhibits HNE2 cell growth by promoting cell cycle arrest and cellular apoptosis. (**A**) Measurement of growth curves of HNE2 cells stably transfected with LOC401317 or an empty vector by MTT assays. LOC401317 overexpression inhibits HNE2 cell growth. Cell cycle distribution (**B**) and apoptosis (**C**) were determined by flow cytometry. LOC401317 arrested HNE2 cell cycling at the G0/G1 phase and induced HNE2 cell apoptosis. Data are expressed as means of 3 independent experiments ± S.D. *P<0.05, **P<0.01, ***P<0.001, relative to control cells.

To elucidate the effect of LOC401317 on the cell growth, an LOC401317 expression vector was stably transfected in HNE2 cells, and the proliferation of these cells was determined by MTT assays. LOC401317 expression significantly inhibited NPC cell growth (Figure 4A). p53 is known to serve as a tumor suppressor by promoting cell cycle arrest and inducting apoptosis, Thus, we evaluated the effect of LOC401317 expression on cell cycle distribution and cell apoptosis in NPC cells by flow cytometry. Cell cycling results indicated that the percentage of the G0/G1-phase cells that overexpressed LOC401317 was significantly higher than that of negative control cells (66.98% vs. 43.84%, *P*<0.01), whereas the percentages of LOC401317 cells in the G2/M and S phases were significantly lower than those of control cells (Figure 4B). These data demonstrated that LOC401317 overexpression induced HNE2 cell cycling arrest at the G0/G1 phase. In addition, we found that the proportion of apoptotic cells significantly increased in LOC401317 transfected HNE2 cells (Figure 4C). LOC401317 thus appears to inhibit HNE2 cell growth both through cell cycle arrest and apoptosis induction. In additional, similar results in other NPC cells (HNE1 and CNE2) were also observed (data not shown).

**Figure 5:**
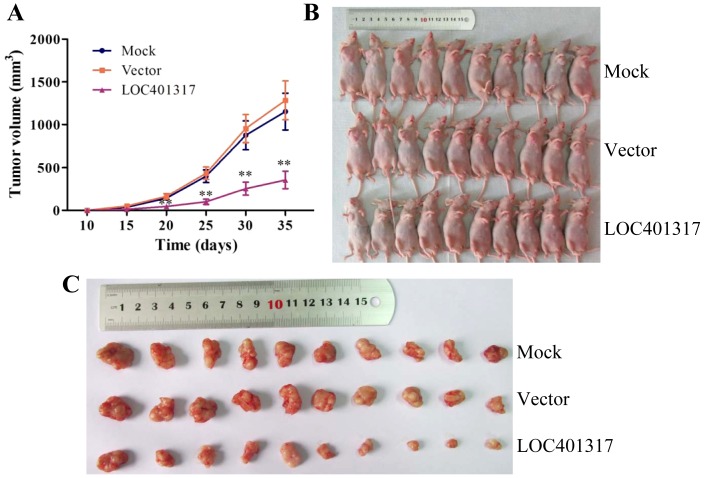
Overexpression of LOC401317 inhibits HNE2 cell growth in xenografted tumors. Untreated HNE2 cells (mock) as well as HNE2 cells that were stably transfected with empty vector (vector) or an LOC401317 overexpression plasmid (LOC401317) were injected subcutaneously into the right flanks of nude mice. (**A**) Xenograft tumor sizes were monitored by measuring every 5 days, between days 10–35 following injection (n = 10 mice per group). Error bars represent SEM, **P<0.01. (**B**) Mice were sacrificed at 35 days post-injection. (**C**) Xenografted tumors were separated and their sizes were measured. Formalin-fixed, paraffin-embedded tissues were prepared from xenografted tumors for subsequent *in situ* hybridization (ISH) or immunohistochemistry (IHC) staining.

We further investigated the tumor suppressive function of LOC401317 *in vivo* using a subcutaneous xenograft-transplanted tumor model in nude mice. Parental control HNE2 cells and HNE2 cells stably transfected with either an empty vector and or the LOC401317 expression plasmid were injected subcutaneously into the right flank of each animal. HNE2 cells expressing LOC401317 showed significantly reduced tumor growth rates (Figure 5A) and final tumor sizes (Figure 5B & C), compared with parental HNE2 cells (mock) and HNE2 cells stably transfected with the empty vector (vector) groups.

### Preliminary investigation of the mechanism by which LOC401317 inhibits NPC cell growth

**Figure 6:**
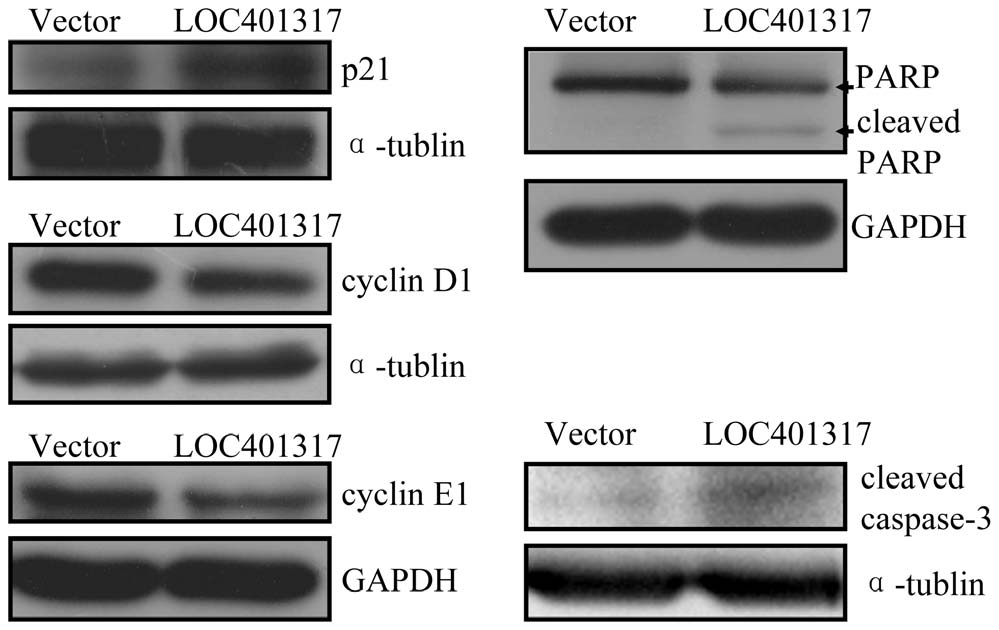
Downstream effector molecules regulated by LOC401317 overexpression in HNE2 cells. Overexpression of LOC401317 increased p21, cleaved PARP, and caspase-3 protein expression, while inhibiting cyclin D1 and cyclin E1 expression. The expression of α-tubulin or GAPDH was detected for protein loading controls.

Because LOC401317 expression arrested HNE2 cell cycling, induced HNE2 apoptosis, and inhibited NPC cell growth *in vitro* and *in vivo*, we proceeded to investigate potential mechanisms to explain how LOC401317 inhibits NPC cell growth. First, we measured p53 and MDM2 expression at both the mRNA and protein levels after overexpression of LOC401317 in HNE2 cells to determine whether LOC401317 feedback inhibits p53 and/or MDM2 expression. qRT-PCR and western blotting results indicated that LOC401317 has no effect on either p53 or MDM2 expression (data not shown). Because LOC401317 can arrest HNE2 cells in the G0/G1 stages and induces apoptosis, we next examined expression levels of key effector molecules of the cell cycle G1/S checkpoint and apoptosis. Western blotting results showed that the expression of p21, cleaved PARP, and caspase-3 were increased, while the cyclin D1 and cyclin E1 were decreased in HNE2/LOC401317 cells, compared to HNE2/vector cells (Figure 6).

**Figure 7:**
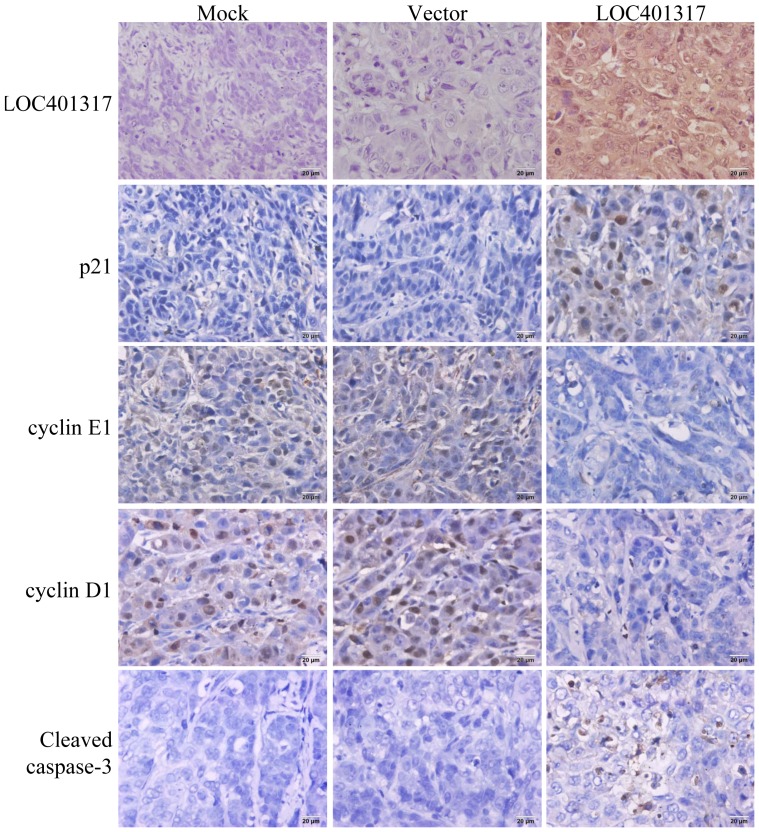
The expression of LOC401317 and downstream effector molecules was validated in xenograft tumor tissues. Xenograft tumor tissues shown in figure 5C were formalin fixed and paraffin embedded. LOC401317 expression was detected by *in situ* hybridization (ISH) using LOC401317-specific probes, while the expression of p21, cyclin E1, cyclin D1, and cleaved caspase-3 were detected by immunohistochemical (IHC) staining. LOC401317 upregulated p21, downregulated cyclin E1 and cyclin D1, and induced caspase-3 cleavage. (magnification: ×400, scale bars: 20 µm).

These results were validated in xenografted tumor samples. LOC401317 expression was confirmed in xenografted tumor samples by *in situ* hybridization (ISH), revealing a strong LOC401317 staining signal representing high LOC401317 expression in xenograft samples derived from HNE2 cells that overexpressed LOC401317. In contrast, the expression of LOC401317 in parental and empty vector-transfected cells was comparatively very low (Figure 7). The expression of p21, cyclin D1, and cleaved caspase-3 was also detected in xenograft tumor tissues by immunohistochemical (IHC) staining. Consistent with our *in vitro* results, higher p21 and cleaved caspase-3 expression and lower cyclin D1 expression were detected in HNE2/LOC401317 xenograft tumors (LOC401317), as compared to those in HNE2/vector (vector) and HNE2/mock tumors. Together, these data indicate that LOC401317 overexpression may arrest the HNE2 cell cycle through p21 and cyclins, while inducing apoptosis through a caspase-dependent mechanism.

## Discussion

lncRNAs were once considered the transcriptional “noise” of the genome; however, recent studies have revealed that lncRNAs govern many important biological functions and have generated significant interest in the field of molecular biology[19]. Aberrant expression or dysfunctional activities of lncRNAs has been discovered in multiple tumor types [25], [38]. Substantial evidence indicates that lncRNAs participate in all steps of tumor initiation and development [25]–[28]. Several studies have shown that lncRNAs are of great importance in the diagnosis and treatment of tumors, and that lncRNAs are useful as novel prognostic tumor biomarkers [37], [39]–[42]. For example, the lncRNA DD3 has been developed into a new diagnostic marker for prostate cancer [39]. HOTAIR may also serve as a potential biomarker for the lymph node metastasis of hepatocellular carcinoma [40]. MALAT1 expression is correlated with the prognosis of non-small cell lung cancer [37]. Additionally, inhibition of HOTAIR expression in liver cancer cell lines increases the chemosensitivity of tumor cells to cisplatin and doxorubicin [41]. Preventing the interaction between HOTAIR and the PRC2 or LSD1 complex may limit the metastatic potential of breast cancer cells [42]. Some lncRNAs have also been demonstrated to be associated with the tumorigenic process of NPC [26], [43], [44]. Zhang *et al.*, found that lncRNA LINC00312 expression is negatively correlated with tumor size but positively correlated with lymph node metastasis in NPC [26]. Nie *et al*. found that lncRNA HOTAIR expression is an independent prognostic marker for NPC progression and survival [43]. Gao *et al*. discovered that some lncRNAs were deregulated in primary and recurrent NPC and that these lncRNAs may play a role in the occurrence and recurrence of the tumor [44].

The tumor suppressor p53, which is encoded by the most frequently mutated gene in human tumors (*TP53*), plays a crucial role in maintaining genomic stability and tumor suppression [12]–[14]. It is known that p53 is involved in the carcinogenesis and tumor progression of many cancers [45], [46]. Recent studies have revealed that lncRNAs interact with the p53 pathway and form a complex regulatory network. p53 also can regulate the expression of many lncRNAs that execute important biological functions [29]–[33]. For example, the regulation of lncRNA loc285194 and lincRNA-p21 by p53 plays a critical role in various types of cancer [32], [33]; however, to our knowledge, regulatory networks between p53 and lncRNAs have not been studied in NPC.

In the present study, we identified 133 upregulated lncRNAs and 1057 downregulated lncRNAs in HNE2 cells overexpressing p53, using an lncRNA expression microarray. These lncRNA molecules may participate in the *TP53* regulation network and play important roles in NPC carcinogenesis; thus, further investigation of their functions is merited. Among these lncRNAs, we chose 5 upregulated lncRNAs for validation studies using qRT-PCR. The most significantly upregulated lncRNA, LOC401317, was selected for further functional studies. Our results showed that LOC401317 was directly regulated by p53 through the p53-binding site adjacent to its potential promoter and overexpression of LOC401317 inhibited HNE2 cell growth by inducing cell cycle arrest and apoptosis *in vivo* and *in vitro*. These results suggest that LOC401317 may be an effective novel target for NPC gene therapy. For example, the restoration of endogenous LOC401317 expression in NPC patients or the introduction of exogenous LOC401317 into tumor cells may inhibit NPC cell proliferation and benefit NPC patients.

Accumulating evidence indicates that lncRNAs participate in many important physiological processes by modulating gene expression at the epigenetic, transcriptional, and post-transcriptional levels [21]–[24]. Mechanisms underlying how lncRNAs regulate gene expression include the induction of chromatin remodeling and histone modification, transcription interference, regulation of alternative splicing patterns, generation of endogenous siRNAs, and modulation of protein activity and localization [47], [48]. Additionally, lncRNAs can interact with microRNAs as competitive endogenous RNAs (ceRNAs) to participate in regulating the expression of target genes [33], [49], [50]. Our preliminary exploration of the mechanisms that LOC401317 utilizes as a tumor suppressor shows that LOC401317 may cause cell cycle arrest by increasing p21 expression and decreasing cyclin D1 and cyclin E1 expression. Our results also suggest that LOC401317 induces apoptosis by a caspase-dependent mechanism. However, the detailed molecular mechanisms by which LOC401317 inhibits NPC cell growth, how LOC401317 regulates downstream p53 signaling pathways, and how LOC401317 expression in NPC samples correlates with the prognosis of NPC patients remain unclear and will be the focus of prospective studies.

## Conclusions

In summary, this study is the first to report the construction of a p53-regulated lncRNA profile in an NPC cell line (HNE2) and identify a set of lncRNAs that are dysregulated by p53 overexpression. Among these p53-regulated lncRNAs, we found that LOC401317 was the lncRNA that was most highly induced by p53 transgene expression and further investigated its function *in vitro* and *in vivo* as a candidate tumor suppressor. Our data demonstrate that LOC401317 is directly transcribed by p53 through a p53-binding site adjacent to its potential promoter, and that LOC401317 overexpression inhibits HNE2 cell growth by arresting cell cycle progression and inducing apoptosis. These results imply that LOC401317 is a novel component of the p53 regulatory network and may be an effective target for NPC therapy.

## Supporting Information

Table S1 **Detailed information regarding p53-regulated lncRNAs and their expression data in HNE2 cells following *TP53* overexpression.**
(XLS)
